# Supplementary material for: 3D mechanical characterization of single cells and small organisms using acoustic manipulation and force microscopy
Source: Nat Commun. 2021 May 10;12:2583. doi: 10.1038/s41467-021-22718-8 (PMC8110787; doi:10.1038/s41467-021-22718-8)
Supplement: Supplementary file 2 — Supplementary Information [file 41467_2021_22718_MOESM2_ESM.pdf]

Supplementary Information

Manuscript:

3D Mechanical Characterization of Single Cells and Small Organisms using Acoustic Manipulation and Force Microscopy

Nino F. Läubli, Jan T. Burri, Julian Marquard, Hannes Vogler, Gabriella Mosca, Nadia Vertti-Quintero, Naveen Shamsudhin, Andrew deMello, Ueli Grossniklaus, Daniel Ahmed, Bradley J. Nelson

Supplementary Figures

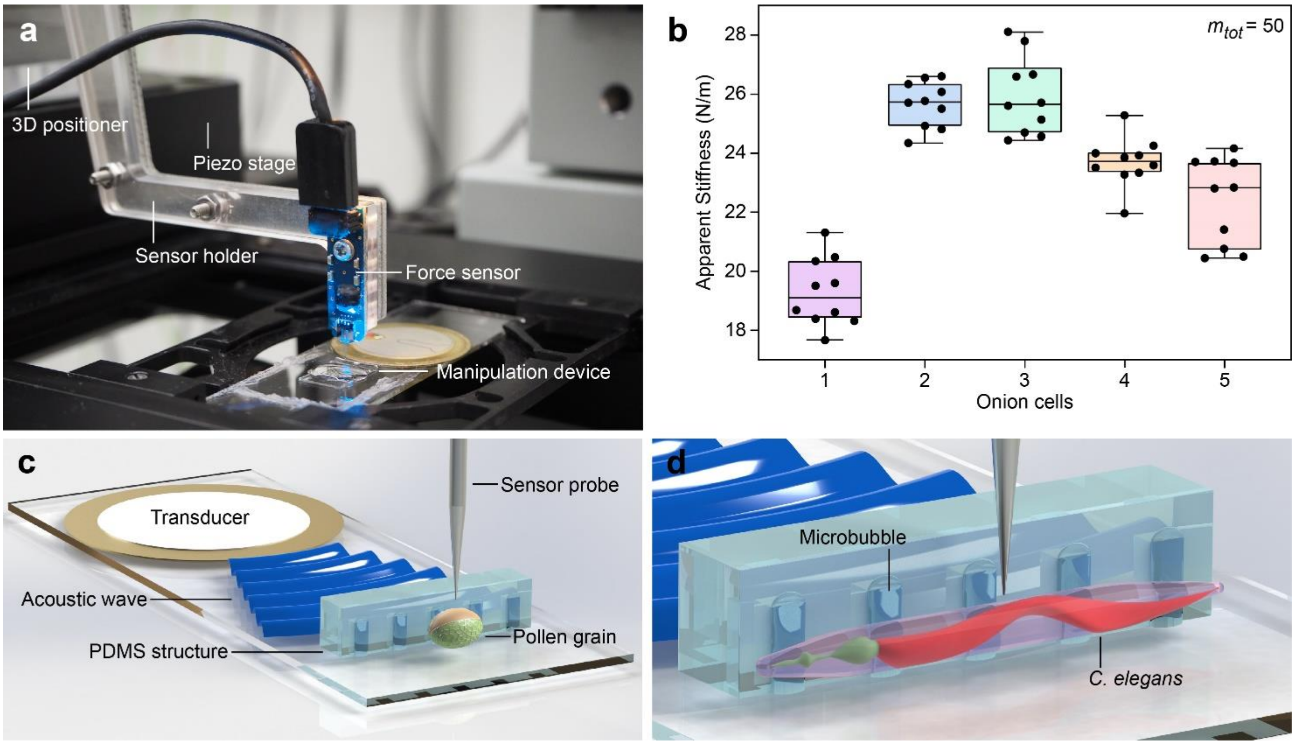

Supplementary Fig. 1| Setup for acoustic manipulation and microindentation.

a) A photograph of the manipulation device integrated into a CFM. The force sensor is moved using a 3D micropositioner. Once the sensor is positioned, the indentation process is performed via the high-precision piezo stage. A technical drawing of the sensor holder as well as the design of the acoustic device can be found on github (see Data Availability). Further information on individual components of the indentation system is provided in Vogler et al.[1] b) Measurements on onion epidermal cells used to quantify the repeatability of our mechanical characterizations of biological specimens. The graph shows the apparent stiffness values for 5 independent onion cells with 10 measurements each. All 10 measurements were performed on the same location of specimen to quantify the repeatability of our characterization method. The boxes represent the interquartile ranges, the center lines represents medians, and the whiskers denote the ranges of minima and maxima. The resulting average coefficient of variation CV is 4.8%. A detailed statistical evaluation is provided in Supplementary Note 2. c) A schematic showing the arrangement of the manipulation device with a pollen grain. The specimen is trapped and re-oriented to the sensor probe using acoustic excitation of microbubbles. d) A detailed visualization with a *Caenorhabditis elegans* nematode in the manipulation device. By exciting multiple parallel microbubbles, the same design as shown for pollen grains allows manipulation of the nematode.

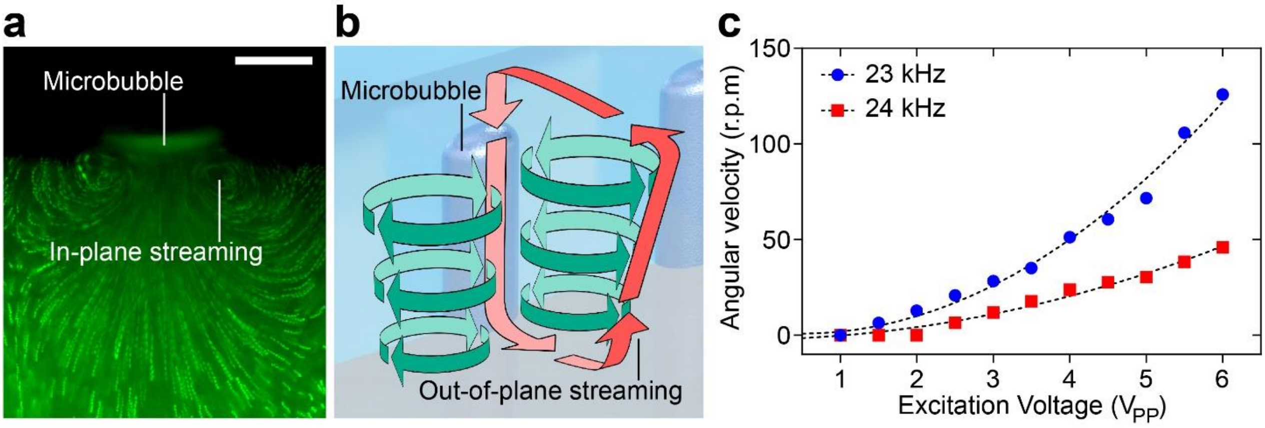

Supplementary Fig. 2| Acoustic streaming and controllability.

a) Counter-rotating, in-plane vortices near the bubble/liquid interface. The streaming is visualized using 1 µm fluorescent particles. b) A 3D schematic illustrating the two co-existing acoustic streaming patterns. The out-of-plane vortices are higher than the microbubble, while the in-plane vortices are limited to the height of the bubble. c) A graph showing the controllability of the out-of-plane rotation for a single lily pollen grain. Its rotational velocity depends on the voltage as well as the frequency used to drive the piezoelectric transducer. Each data point represents a single measurement for angular velocity of a pollen grain at a specific excitation frequency and voltage. For both data sets, i.e., at 23 and 24 kHz, the rotation of a single pollen was characterized to prevent noise based on biological variation.

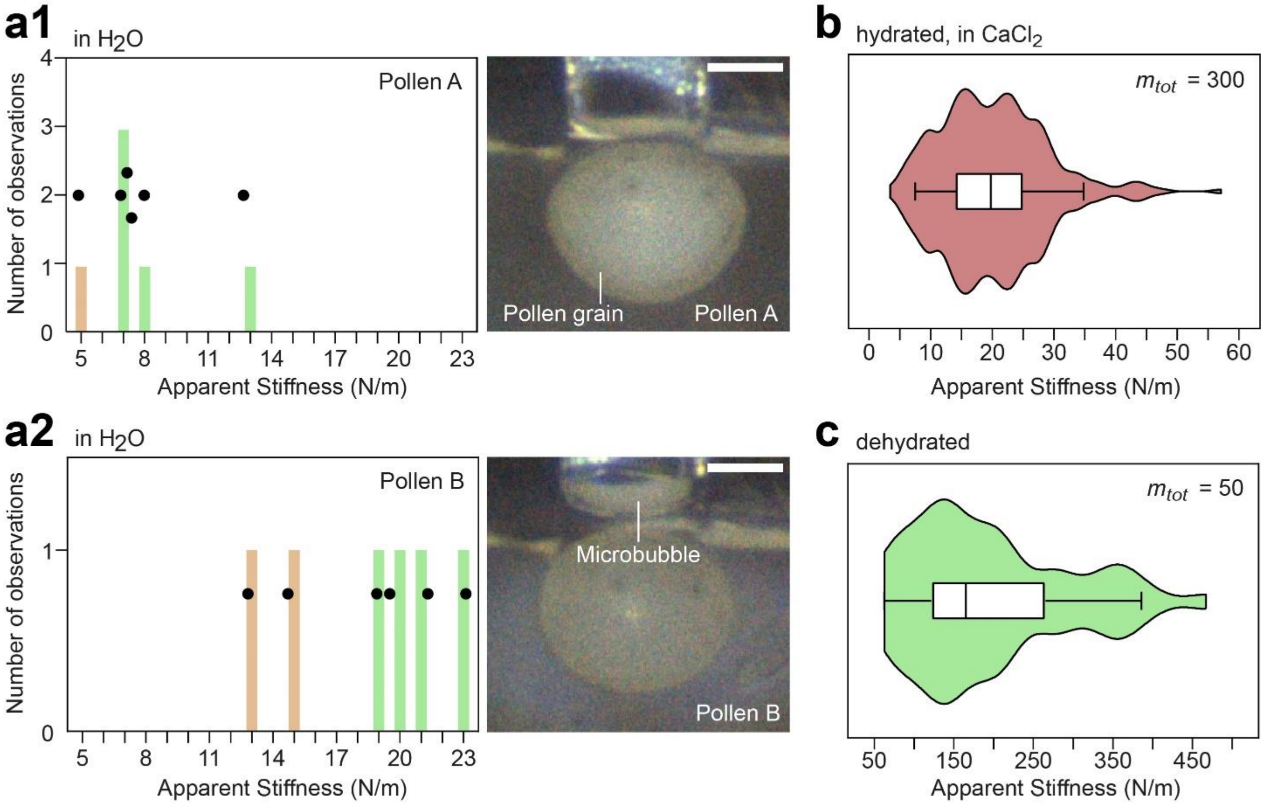

Supplementary Fig. 3| Stiffness variations of lily pollen grains.

a) No correlation between stiffness and shape or size of the pollen grains was observed. The hydrated pollen grains in a1) and a2) are both comparable in dimension; however, their mechanical properties vary strongly (intine, brown; exine, green). The pollen grains were characterized on the same date using the same force sensor and were taken from the same flower sample. A violin plot containing the apparent stiffness values for intine and exine (m = 300) measured on 30 biologically independent lily pollen grains in $CaCl_2$ solution. On each sample, 10 independent measurements to quantify the different surface regions. The box represents the interquartile range, the center line represents the median, and the whiskers represent the 5th and 95th percentiles. The maxima and minima are denoted by the start and end of the violin plots. c) A violin plot containing the stiffness values characterized through 50 independent single indentations on 50 biologically independent dehydrated lily pollen grains. The indentations have only been made on the exine, as the intine was inaccessible due to the folded state of the pollen. An average apparent stiffness value of 194.2 N/m (95% confidence interval [166.7 N/m, 222.7 N/m]) was detected for the exine of non-hydrated pollen grains. The box represents the interquartile range, the center line represents the median, and the whiskers represent the 5th and 95th percentiles. The maxima and minima are denoted by the start and end of the violin plots. Scale bars = 50 µm.

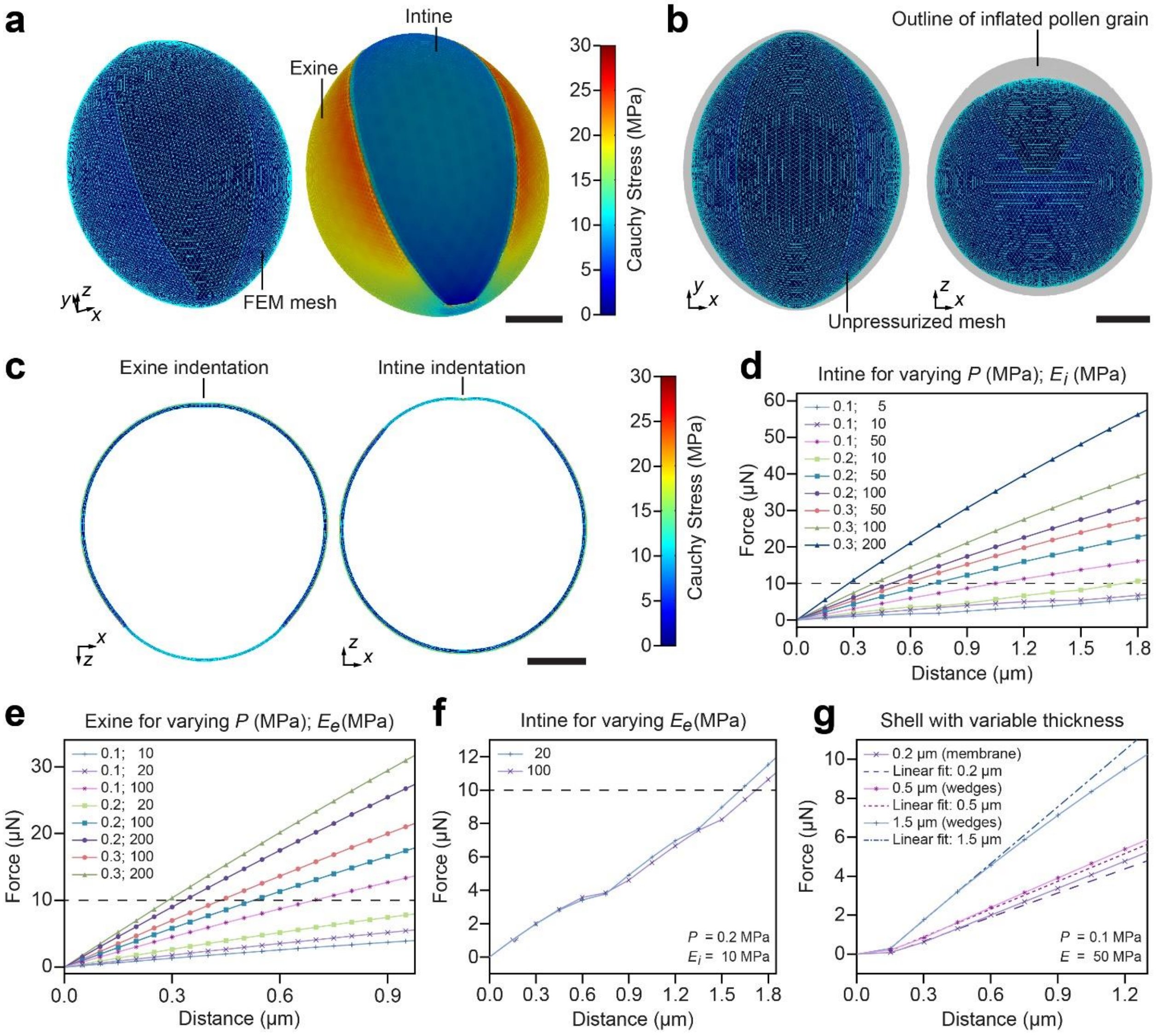

Supplementary Fig. 4| FEM simulation of mechanical pollen characterization.

a) Simulated unpressurized lily pollen grain next to the inflated version with turgor pressure P = 0.2 MPa. b) FEM mesh of an unpressurized lily pollen grain as well as, for comparison, the outline of an inflated pollen grain with turgor pressure P = 0.2 MPa, Poisson's ratio v = 0.3, Young's modulus of intine E_i = 10 MPa, and Young's modulus of exine E_e = 100 MPa. c) Indentation simulation on the exine and intine of an inflated pollen grain. The position of the indentation is indicated by black lines. d) Indentation of intine for different combinations of turgor pressure P and intine Young's moduli E_i. The dashed line denotes the maximum force applied during experimental characterization. e) Indentation of the exine for varying turgor pressure P and exine Young's moduli E_e. For each measurement, the intine Young's modulus E_i is half of the corresponding exine Young's modulus E_e. f) Indentation of the colpus (intine) for varying exine Young's moduli E_e. The calculated apparent stiffness for each configuration in d), e), and f) can be found in Supplementary Table 1. g) Indentation of a spherical shell model (diameter = 100 µm, Poisson's ratio v = 0.3, Young's modulus E = 50 MPa, turgor pressure P = 0.1 MPa) to investigate the source of the simulated sublinear indentation curves for the pollen grain. The shell thickness was varied across 0.5 and 1.5 µm (wedges simulation) and set to 0.2 µm for the membrane simulation. As can be seen, for the same material properties (pink star and blue plus), decreasing material thickness can affect the indentation curve, shifting it from a sublinear to superlinear behavior. Each linear fit has been performed in an indentation depth range of 0.1 to 0.5 µm. Scale bars = 25 µm.

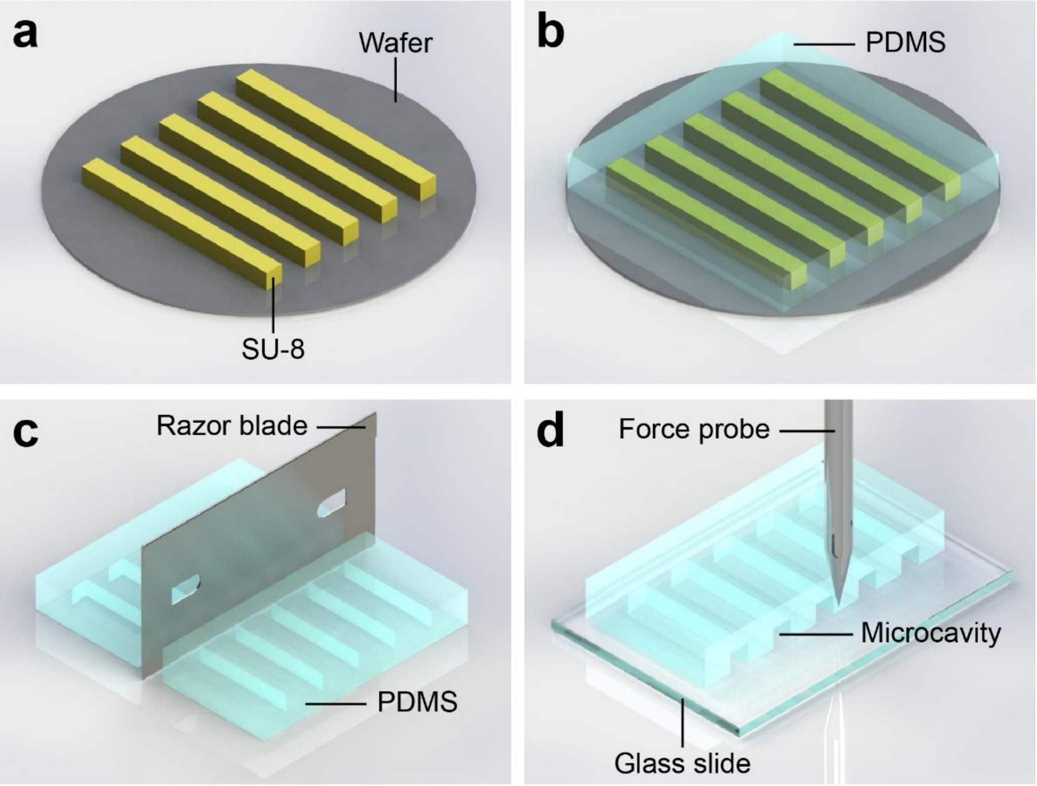

Supplementary Fig. 5| A schematic showing the fabrication steps for microcavities.

a) Microchannels are fabricated using SU-8-based photolithography. b) The microchannels are transferred into PDMS by molding. c) The PDMS is cut perpendicular to the microchannels to create microcavities. d) By oxygen plasma bonding, the PDMS is chemically fixed to a glass slide to seal the bottom of the microcavities. Thus, one side remains open allowing for the trapping of microbubbles and subsequent manipulation of the specimen.

Supplementary Tables

Supplementary Table 1| Simulated intine and exine stiffness for varying turgor pressure and Young's moduli.

All simulations assume a constant poisson ratio of 0.3 as well as constant cell wall thicknesses, i.e., 1.5 µm for the intine and 0.5 µm for the exine. The apparent stiffness values k_i and k_e have been calculated through linearization between indentation distance 1.5 and 1.8 µm and 0.4 and 0.8 µm, respectively. Please note that combinations of higher pressure and lower material stiffness (for the intine) led to numerical failure of the model. However, we do not consider such combinations as likely because Young's moduli smaller than 1 MPa have never been reported for plant tissues and the induced stretch would have been high and would produce very distinct curvatures for such layers. [1] denotes the apparent stiffness for the simulations shown in Supplementary Fig. 4d. [2] denotes the apparent stiffness values of the indentation simulations shown in Fig. 2j.

Model Parameters			Apparent Stiffness (N/m)	
Turgor Pressure P (MPa)	Young's Modulus Intine E_i (MPa)	Young's Modulus Exine E_e (MPa)	Intine k_i @ 1.65 µm	Exine k_e @ 0.6 µm
0.1	5	10	4.26	3.98
0.1	10	20	4.36	5.62
0.2	10	20	8.52 [1]	7.98
0.2	10	50	8.23	10.6
0.2	10	100	7.9 [1, 2]	12.7 [2]
0.1	50	100	7.82	13.69
0.2	50	100	11.03	17.96
0.3	50	100	12.17	21.7
0.2	100	200	15.65	27.37
0.3	100	200	19.29	31.82
0.3	200	400	26.93	49.95

Supplementary Notes

Supplementary Note 1| Reynolds number for an oscillating microbubble

According to P. Marmottant and S. Hilgenfeldt,[2] the Reynolds number for an acoustically excited microbubble can be calculated as:

$$\mathrm{Re} = \varepsilon^2 \left(\frac{2\pi f a^2}{\nu}\right)^{\frac{1}{2}} \approx 0.35,$$

with the normalized oscillation amplitude ε = 0.10, the bubble radius $a \approx 100$ µm, the excitation frequency $f = 20$ kHz, and the kinematic viscosity of water $\nu = 1.0x10^{-6}$ m^2/s. The actual value may differ given the requirement of sphericity.

Supplementary Note 2| Repeatability of mechanical characterization of biological specimens

To quantify the repeatability of our indentation-based mechanical characterizations, 50 measurements were performed on 5 onion epidermal cells, i.e., 10 repeated indentations per cell (see Supplementary Fig. 1b). Onion epidermal cells were chosen as they have a homogeneous cell wall and prevent possible measurement artifacts through realignment or slippage. Repeatability for each sample was derived through the coefficient of variation (CV) according to B. Carstensen.[3] The individual CVs for the cells 1 – 5 are 6%, 3%, 5.1%, 3.5%, and 6.6%, respectively, which leads to an average CV of 4.8%.

It is crucial to highlight that the reproducibility of mechanical characterizations is also affected by changes in the biological specimen, such as local cell wall weakening, induced through the repeated indentations or changes in turgor pressure due to the hydration state of the sample.

Supplementary Note 3| Detailed statistical normality test

Given the near bimodal nature of the obtained apparent stiffness values for pollen grains, all data has been tested for normality using a D’Agostino omnibus K2 prior to the statistical evaluation through t-tests. Intine apparent stiffness (K2 = 14.2, $p = 0.0008$) as well as exine apparent stiffness (K2 = 11.47, $p = 0.0032$) from pollen in deionized water were found to show a non-normal distribution. Combined intine as well as exine measurements obtained from lily pollen grains in deionized water have been detected as not normally distributed (K2 = 17.95, $p = 0.0001$). Combined intine as well as exine measurements obtained from lily pollen grains in $CaCl_2$ solution were found to show a non-normal distribution (K2 = 33.85, $p < 0.0001$). Normality tests for apparent stiffness ratios k_i/k_e from lily pollen grains in deionized water (K2 = 0.3949, $p = 0.8208$) and pollen grains in $CaCl_2$ solution (K2 = 2.189, $p = 0.3347$) are not significant, i.e., the data can be treated as normally distributed. Measurements from folded lily pollen grains have been found normally distributed (K2 = 5.703, $p = 0.0578$), albeit the near-significance of the result leaves room for discussion.

Please note that t-tests have only been applied to stiffness ratios, which do not display bimodality and can be assumed as sampled from a Gaussian distribution.

Supplementary Note 4| Detailed statistical comparison of stiffness ratios

A two-tailed t-test for independent-samples and unequal variances (F = 4.79, p = 0.03) was performed to compare the stiffness ratios in deionized water (M = 0.56, SD = 0.12) and calcium chloride solution (M = 0.66, SD = 0.08). The difference, -0.1, BCa 95% with a confidence interval [-0.15, -0.05], was significant t[58] = -3.84, p = 0.000312.

Supplementary Note 5| Detailed statistical comparison of stiffness regions in *C. elegans*

The stiffness values of the two regions were compared with a two-tailed t-test for independent samples with equal variances (F = 2.85, p = 0.09) and the difference, 0.22, BCa 95% confidence interval [0.16, 0.28], was reported significant with t[48] = 7.89 and p = 3.14e-10.

Supplementary Note 6| Numerical simulation of pollen grain indentation

Indentation simulations were performed with the software MorphoMechanX (www.morphomechanx.org) and used the finite element method (FEM) to model pollen grain mechanics.

The pollen mesh was generated by extruding to four wedge layers (each of 0.5 µm thickness) a rotation ellipsoid represented by a membrane mesh whose major and minor axes were, respectively, 128 µm and 97 µm. To represent the colpus region, where only the intine layer is present, a portion of the outer layer of wedges was manually selected and eliminated to produce the final mesh represented in Supplementary Fig. 4a. The average in-plane element length is 2 µm.

We used a hyperelastic Sant-Venant Kirchhoff isotropic material law for the whole grain, characterized by a Young's modulus E (connected to the material stiffness) and a Poisson ratio ν (defining the tendency of the material to preserve its volume; in our case, this parameter was ν = 0.3, making the material quite incompressible). The three inner wedge layers represent the intine and are assigned the same material properties, while the outer layer, where present, represents the exine and is assigned different material properties as described in the main text.

The turgor pressure P was assigned on the internal faces of the grain and, when mechanical equilibrium is computed, this will inflate the pollen grain (see Supplementary Fig. 4a, b).
To compute the mechanical equilibrium, for both the inflation and indentation processes, a FEM-based simulaltion was used. The nodal displacement over the mesh coordinates was obtained through an iterative semi-implicit Euler method (see Vetterling et al.[4]):

$$\mathbf{u}_{\mu i}(t_k + 1) = \mathbf{u}_{\mu i}(t_k) + dt_k \left(1 - dt_k \frac{\partial \Pi}{\partial \mathbf{u}_{\mu i} \partial \mathbf{u}_{\theta j}}\right)^{-1} \frac{\partial \Pi}{\partial \mathbf{u}_{\theta j}} \qquad (6.1)$$

where $\mathbf{u}_{\mu i}$ indicates the nodal displacement of the mesh node μ from the reference configuration in the 3D space coordinate i (Greek letter indicates always nodal indexes, Latin letter the space coordinate). $\Pi(\boldsymbol{u})$ indicates the total potential energy function, which is composed of the strain energy function and the external forces function. For an isotropic Saint Venant-Kirchhoff material, $\Pi(\mathbf{u})$ can be represented as follows:

$$\Pi(\mathbf{u}) = \int_{\Omega_0} \frac{\lambda}{2}\,\mathrm{Tr}\left(\boldsymbol{\epsilon}(\mathbf{u})\right)^2 + \mu\,\mathrm{Tr}\left(\boldsymbol{\epsilon}(\mathbf{u})^2\right)\,dxdydz \; + P\int_{\tilde{\Omega}} dx\,dydz \qquad (6.2)$$

The first integral refers to the strain energy component and is given by the body deformation (but it is invariant for rigid body deformations), λ and μ are the Lamé coefficients, which are related to the Youngs Modulus E and the Poison ratio ν in the following way:

$$\lambda = \frac{E\nu}{(1+\nu)(1-2\nu)}, \qquad \mu = \frac{E(1-\nu)}{(1+\nu)(1-2\nu)} \qquad (6.3)$$

The Green-Lagrange strain tensor is represented by $\boldsymbol{\epsilon}$, Tr indicates the trace of the tensor and the integral is to be intended over the whole continuum body volume. The second integral refers to the contribution of pressure to the total potential energy, the volume integral refers to the empty space enclosed by the surface of the body exposed to pressure (for a pollen grain, this refers to the hollow volume inside the grain). This formulation is valid for large strains and large deformations.

In the forces equilibrium computation (which dictates the body deformation as in Eq. 6.1), the first and second derivatives of the total potential energy are computed with respect to the nodal coordinates. Such computation is performed element-wise and then the resulting contribution on each node is assembled globally. With respect to a single element, the continuous displacement variable $\mathbf{u}(x, y, z)$ is, through multi-linear basis functions, represented as:

$$\mathbf{u}(x, y, z) = \sum_{\mu=1}^{6} \hat{\mathbf{u}}_{\mu}\,\phi_{\mu}(\xi, \eta, \zeta) \qquad (6.4)$$

where $\hat{\mathbf{u}}_{\mu}$ stands for the vectorial nodal displacement at the element node μ, while the multi-linear basis functions ϕ_{μ} are expressed with respect to the wedge-isoparametric coordinates, which represent a wedge made by two parallel isosceles right-angled trianglular faces connected by three squares of side length 1 unit.

$$\xi = \xi(x, y, z), \quad \eta = \eta(x, y, z), \quad \zeta = \zeta(x, y, z) \qquad (6.5)$$

ξ and η define the wedge triangular faces, so that $0 \leq (\xi, \eta) \leq 1$ and $(\xi + \eta) \leq 1$, while $-1 \leq \zeta \leq 1$. A representation of the multilinear basis function can be found at:[5] *https://help.febio.org/FEBio/FEBio_tm_2_7/FEBio_tm_2-7-Subsection-4.1.2.html* and integral quantities are computed using a six-point Gaussian quadrature rule as again shown at the above mentioned website.

For a more detailed derivation, see Mosca *et al.*[6] Convergence is reached when the residual (given in our case by the average of the minimum and maximum of the norm of the derivative of the total potential energy as computed at each mesh node) is below the threshold 10e-6.

After pressurization convergence is achieved, the indentation process occurs. This has been modeled in a way analogous to that described in Mosca *et al.*[7] The only difference being that the indented node belongs to a wedge and not a membrane element.

The model supports an automated indentation cycle where material parameters for the pollen grain (independently for exine and intine) and the turgor pressure can be varied as prescribed in a list and the respective indentation curves are saved. The stiffness is computed from these curves and recorded into a csv file by a python script available on Github (see Code Availability).

References

1. Vogler, H., Burri, J. T., Nelson, B. J. & Grossniklaus, U. Simultaneous measurement of turgor pressure and cell wall elasticity in growing pollen tubes. in *Methods in Cell Biology* (2020). doi:10.1016/bs.mcb.2020.04.002.

2. Marmottant, P. & Hilgenfeldt, S. Controlled vesicle deformation and lysis by single oscillating bubbles. *Nature* **423**, 153–156 (2003).

3. Carstensen, B. Repeatability, Reproducibility and Coefficient of Variation. in *Comparing Clinical Measurement Methods* 107–114 (John Wiley & Sons, Ltd, 2010). doi:10.1002/9780470683019.ch9.

4. Vetterling, W. T., Press, W. H., Teukolsky, S. A. & Flannery, B. P. *Numerical Recipes Example Book (C++): The Art of Scientific Computing*. (Cambridge University Press (CUP), 2002).

5. FEBio Software. Pentahedral Elements. https://help.febio.org/FEBio/FEBio_tm_2_7/FEBio_tm_2-7-Subsection-4.1.2.html.

6. Mosca, G., Lane, B., Strauss, S., Runions, A. & Smith, R. 3D Mechanical modeling of multicellular biomechanics & growth, controlled by gene expression with MorphoMechanX. Manuscript in Preparation.

7. Mosca, G., Sapala, A., Strauss, S., Routier-Kierzkowska, A.-L. & Smith, R. S. On the micro-indentation of plant cells in a tissue context. *Phys. Biol.* **14**, 015003 (2017).
