# Supplementary material for: 3D mechanical characterization of single cells and small organisms using acoustic manipulation and force microscopy
Source: Nat Commun. 2021 May 10;12:2583. doi: 10.1038/s41467-021-22718-8 (PMC8110787; doi:10.1038/s41467-021-22718-8)
Supplement: Supplementary file 7 — Reporting Summary [file 41467_2021_22718_MOESM7_ESM.pdf]

Corresponding author(s): Daniel Ahmed
Last updated by author(s): Mar 12, 2021

Reporting Summary

Nature Research wishes to improve the reproducibility of the work that we publish. This form provides structure for consistency and transparency in reporting. For further information on Nature Research policies, see our Editorial Policies and the Editorial Policy Checklist.

Statistics

For all statistical analyses, confirm that the following items are present in the figure legend, table legend, main text, or Methods section.

n/a	Confirmed	
☐	☒	The exact sample size (n) for each experimental group/condition, given as a discrete number and unit of measurement
☐	☒	A statement on whether measurements were taken from distinct samples or whether the same sample was measured repeatedly
☐	☒	The statistical test(s) used AND whether they are one- or two-sided<br>*Only common tests should be described solely by name; describe more complex techniques in the Methods section.*
☒	☐	A description of all covariates tested
☐	☒	A description of any assumptions or corrections, such as tests of normality and adjustment for multiple comparisons
☐	☒	A full description of the statistical parameters including central tendency (e.g. means) or other basic estimates (e.g. regression coefficient) AND variation (e.g. standard deviation) or associated estimates of uncertainty (e.g. confidence intervals)
☐	☒	For null hypothesis testing, the test statistic (e.g. F, t, r) with confidence intervals, effect sizes, degrees of freedom and P value noted<br>*Give P values as exact values whenever suitable.*
☒	☐	For Bayesian analysis, information on the choice of priors and Markov chain Monte Carlo settings
☒	☐	For hierarchical and complex designs, identification of the appropriate level for tests and full reporting of outcomes
☒	☐	Estimates of effect sizes (e.g. Cohen's d, Pearson's r), indicating how they were calculated

Our web collection on statistics for biologists contains articles on many of the points above.

Software and code

Policy information about availability of computer code

Data collection	Olympus CellSense Entry v2.3, Labview 2018, and MorphomechanX ba3c1c73 (Code: www.doi.org/10.5281/zenodo.4589094)
Data analysis	Matlab R2020a (Code: www.doi.org/10.5281/zenodo.4585392), Microsoft Excel 2016, IBM SPSS Statistics 24, and GraphPad Prism 8

For manuscripts utilizing custom algorithms or software that are central to the research but not yet described in published literature, software must be made available to editors and reviewers. We strongly encourage code deposition in a community repository (e.g. GitHub). See the Nature Research guidelines for submitting code & software for further information.

Data

Policy information about availability of data

All manuscripts must include a data availability statement. This statement should provide the following information, where applicable:

- Accession codes, unique identifiers, or web links for publicly available datasets
- A list of figures that have associated raw data
- A description of any restrictions on data availability

The authors declare that data supporting the findings of this study are available within the paper and its supplementary information. Source data are provided with this paper and are, additionally, available on Github together with technical drawings and information on the design of the acoustic device: https://github.com/laeublin/3D-Indentation
Further data can be accessed through the authors.

Field-specific reporting

Please select the one below that is the best fit for your research. If you are not sure, read the appropriate sections before making your selection.

- [x] Life sciences
- [ ] Behavioural & social sciences
- [ ] Ecological, evolutionary & environmental sciences

For a reference copy of the document with all sections, see nature.com/documents/nr-reporting-summary-flat.pdf

Life sciences study design

All studies must disclose on these points even when the disclosure is negative.

Sample size	A sample size of 16 specimens per experimental group is necessary (Dhand, N. K., & Khatkar, M. S. (2014). Statulator: An online statistical calculator. Sample Size Calculator for Comparing Two Independent Means. Accessed 12 March 2021 at http://statulator.com/SampleSize/ss2M.html). In our experiments, a sample size of 30 pollen grains per experimental group was chosen to ensure accurate evaluation and statistical relevance.
Data exclusions	Data containing artifacts (such as noise due to slipping) were removed from all experiments prior to inclusion into the evaluation and interpretation to prevent inaccurate measurements.
Replication	The data presented is based on secondary experiments. The findings and range of the data for both sets of experiments are similar, the experiment was reproducible. Additionally, repeatability of mechanical characterization was demonstrated (average CV = 4.83%).
Randomization	Pollen grains were randomly attributed to water and calcium chloride solution. No allocation was necessary for onion epidermal cells and C. elegans (single experimental groups). All experiments were performed an random samples.
Blinding	All data collection and data evaluation was performed with the same code and independent of experimental groups to ensure equal treatment. Blinding was not relevant for the demonstration of the characterization capability of the manipulation device.

Reporting for specific materials, systems and methods

We require information from authors about some types of materials, experimental systems and methods used in many studies. Here, indicate whether each material, system or method listed is relevant to your study. If you are not sure if a list item applies to your research, read the appropriate section before selecting a response.

Materials & experimental systems

n/a	Involved in the study	
[x]	[ ]	Antibodies
[x]	[ ]	Eukaryotic cell lines
[x]	[ ]	Palaeontology and archaeology
[ ]	[x]	Animals and other organisms
[x]	[ ]	Human research participants
[x]	[ ]	Clinical data
[x]	[ ]	Dual use research of concern

Methods

n/a	Involved in the study	
[x]	[ ]	ChIP-seq
[x]	[ ]	Flow cytometry
[x]	[ ]	MRI-based neuroimaging

Animals and other organisms

Policy information about studies involving animals; ARRIVE guidelines recommended for reporting animal research

Laboratory animals	The investigated nematodes were hermaphrodite, day-2 adult C. elegans of the strain TJ375 (Phsp-16.2::GFP).
Wild animals	The study did not involve wild animals.
Field-collected samples	The study did not involve field-collected samples.
Ethics oversight	No ethical approval was required for our work with pollen grains, onion epidermal cells, and C. elegans.

Note that full information on the approval of the study protocol must also be provided in the manuscript.
