# Supplementary material for: 3D mechanical characterization of single cells and small organisms using acoustic manipulation and force microscopy
Source: Nat Commun. 2021 May 10;12:2583. doi: 10.1038/s41467-021-22718-8 (PMC8110787; doi:10.1038/s41467-021-22718-8)
Supplement: Supplementary file 8 — Description of Additional Supplementary Files [file 41467_2021_22718_MOESM8_ESM.pdf]

Title: Supplementary Movie 1

Description: Out-of-plane rotation of an auto-fluorescent pollen grain. A video of a slow and stable out-of-plane rotation of a single lily pollen grain. The movie is in real-time. The slow angular velocity prevents blur during (auto-) fluorescence imaging.

Title: Supplementary Movie 2

Description: In-plane vortices. A video showing two counter-rotating in-plane vortices near the microbubble/liquid interface. The streaming is visualized using 1 μm fluorescent beads. The movie is in real-time.

Title: Supplementary Movie 3

Description: Animation of the experimental procedure. An animation showing the manipulation and mechanical characterization, i.e., through indentation, of a pollen grain. Acoustic excitation is used to rotate the specimen, while the motion is stopped to measure the mechanical properties. This procedure is repeated to quantify the different areas of the pollen grain.

Title: Supplementary Movie 4

Description: Out-of-plane rotation of a fluorescent C. *elegans* nematode. The video shows a controlled out-of-plane rotation of a fluorescently labelled C. *elegans* worm. The intestine (fluorescent) as well as the distribution of the eggs/embryos (black discs) can be observed throughout the rotation. The movie is in real-time.
